# The ripple effect: a digital intervention to reduce suicide stigma among farming men

**DOI:** 10.1186/s12889-020-08954-5

**Published:** 2020-05-29

**Authors:** Alison J. Kennedy, Susan A. Brumby, Vincent Lawrence Versace, Tristan Brumby-Rendell

**Affiliations:** 1 Deakin University/National Centre for Farmer Health, 75 Pigdons Road, Waurn Ponds, VIC 3216 Australia; 2 Deakin Rural Health, Deakin University, 75 Pigdons Road, Waurn Ponds, VIC 3216 Australia; 3 National Centre for Farmer Health, Western District Health Service, PO Box 283, Hamilton, VIC 3300 Australia

**Keywords:** Suicide stigma, Suicide literacy, Farmer health, Rural health, Digital intervention

## Abstract

**Background:**

Compared with the general population, Australian farmers—particularly men—have been identified as at greater risk of suicide. A complex range of factors are thought to contribute to this risk, including the experience of Stigm*a. stigma* also impacts those who have attempted suicide, their carers, and those bereaved by suicide—manifesting as shame, guilt, social isolation, concealment of death, reduced help seeking and ongoing risk of suicide. This paper evaluates the effectiveness of an intervention, tailored for the farming context, designed to reduce stigma among farming men with a lived experience of suicide.

**Methods:**

The digital intervention used an adult learning model providing opportunity to share insights, reflect, learn and apply new knowledge among people with shared farming interests, suicide experience and cultural context. A range of content—tailored to the gender, farming type and suicide experience of participants—included video stories, postcard messages, education and personal goal setting. Pre- and post- assessment of suicide stigma and literacy was complemented by qualitative data collection during the intervention and participant feedback surveys.

**Results:**

The intervention was successful in reaching members of the target group from across Australia’s rural communities—with diverse geographic locations and farming industries represented. One hundred and sixty-nine participants from the target group (farming males aged 30–64 years) were recruited. While the Stigma of Suicide Scale failed to identify a reduction in self- or perceived-stigma, qualitative data and participant feedback identified behavioural indicators of stigma reduction. Four subthemes—‘growth’, ‘new realisations’, ‘hope’ and ‘encouragement’—highlighted attitudinal and behaviour change indicative of reduced stigma associated with mental health and suicide.

Participants’ baseline suicide literacy (Literacy of Suicide Scale) was high when compared with previous community samples and total literacy scores did not demonstrate significant improvement over time, although literacy about the link between suicide and alcoholism did significantly improve.

**Conclusions:**

These results highlight opportunities in groups with high suicide literacy for targeted stigma reduction and suicide prevention efforts for both the target group and other populations within Australia and internationally. Results also highlight the need to reassess how stigma change is understood and evaluated across a wider range of population groups.

**Trial registration:**

This research project was registered with the Australian New Zealand Clinical Trials Registry (ANZCTR) (ACTRN12616000289415) on 7th March, 2016.

## Background

The risk of suicide significantly increases in rural and remote Australia—particularly for males [1]—with the rate of suicide between 2011 and 2015 suggesting a growing urban-rural divide with sharper increases in death rates outside of major cities [2]. This is despite similar prevalence of diagnosed mental health conditions across metropolitan and rural areas [3]. Farming communities, in particular, have regularly reported a higher risk of suicide—up to twice the rate of the general population—and that this will vary regionally [4–7]. Consistent with general rural suicide rates, is that male farmers are at the greatest risk [5].

A range of factors has been reported as contributing to suicide in farming communities including economic insecurity, unpredictable seasonal conditions, and an uncertain future [8]. Within the social, geographical, and psychological context of Australia’s rural farming communities, there is also recognised stigma related to suicide and help-seeking [9]. Stigma can arise in the form of ‘perceived-stigma’ (a person’s beliefs about negative views that other people have) and ‘self-stigma’ (negative or stigmatised views a person holds about themselves) [10]. Stigma can manifest from lack of knowledge or misinformation, cultural attitudes and discriminating behaviour. In rural Australia, geographic isolation, traditional gender and cultural expectations, and close-knit communities can constrain open discussion about mental health and suicide, and reinforce the effects of stigma [9]. Within this context, the avoidance of emotional vulnerability—combined with feelings of weakness, shame, guilt, selfishness, and the sense of rejection associated with the experience of suicide—can be damaging [11, 12]. Concealment of behaviour and avoidance of help-seeking resulting from stigma may have life-altering effects [13, 14]. General population samples in areas of high suicide have been identified as experiencing increased stigma associated with psychological problems [15].

The culture and context of Australia’s rural and farming communities foster self-reliance, and acclimatisation to risk-taking behaviour, and stoicism [9, 16]. These characteristics have been associated with an increased risk of suicide and the experience of stigma among those who have attempted suicide or are bereaved by suicide [11, 17, 18]. Such stigma is associated with ongoing suicide ideation and complicated grief [15, 19, 20]. Within the intertwined social relationships of rural communities, the impact of both suicide and stigma can be particularly profound and long lasting. It is therefore important to understand how stigma is constructed, experienced and expressed, its consequences, and how it may be overcome.

### The effect of stigma

Stigma is a noted barrier to individuals engaging support and articulating suicide ideation [14, 20]. Stigma reduces contact with trained professionals, particularly when prior contact attempts have had negative consequences or been unhelpful [17, 20]. The stigma of seeking professional mental health support—compounded by inequitable access to health services, and a determination to solve one’s own problems—often deters farmers (male and female) from seeking assistance [12, 16, 21]. Adding to this is the fear within farming communities of being judged negatively, considered weak or perceived as untrustworthy following an expression of emotional pain [9].

Social withdrawal due to perceived negative judgement increases the risk of psychological distress and reduces the protection from vulnerability that existing social support networks can provide [22, 23]. Regardless of whether there is evidence of social exclusion by the community or this is inaccurately perceived, the ramifications for already emotionally vulnerable and geographically isolated people could be significant.

When suicide stigma is experienced, concealment of suicide as the cause of death [24] and reduced reporting of suicide may occur [25]. This is particularly pertinent within the social context of farming communities, where anonymity is low and suicide stigma has been identified [17, 24].

A lived experience of suicide significantly increases the ongoing risk of suicide and the likelihood of poor mental health outcomes [19, 20, 26]. Stigma further increases the risk of suicide for those already suffering psychologically [15].

### Stigma reduction efforts

To date, efforts to decrease stigma have largely been focussed on reducing the negative perceptions associated with living with a mental illness. Encouraging disclosure and social connection, attitudinal change, improved awareness and empowerment through knowledge have been identified as effective methods of self- and perceived-stigma reduction [27–29]. Mental illness stigma research further suggests that combining education and contact with persons living with a mental health condition can be effective in reducing stigma [30]. In summary, increasing mental health literacy has been determined as an effective means of reducing mental health stigma.

### What is known about reducing suicide stigma

Only recently has research focus turned specifically to suicide stigma [31–37]. To date, there has been limited success in reducing stigma associated with suicide and suicide bereavement [38], and some international evidence suggesting that community suicide stigma is reducing [39]. Whether this extends to the experience of self- and perceived-stigma, and covers the range of suicide experience is less clear (e.g., bereavement, suicide attempt, suicide ideation, and caring for someone experiencing suicidality). While some attempt has been made to understand suicide stigma in rural Australia [40], there remains an absence of evidence and effort to specifically target suicide stigma in farming populations. This is despite the evidence of heightened risk of suicide in farming populations, along with cultural and contextual factors that suggest stigma—and its negative effects—may be more profoundly experienced in this population. This lack of evidence was considered in the development of the Ripple Effect digital intervention undertaken by Kennedy and colleagues [41]. This manuscript now evaluates the effectiveness of the digital intervention to:

1. Reduce self-stigma and perceived-stigma among men—aged 30–64 years, from Australian rural farming communities—with a lived experience of suicide, as shown by validated assessment tools and qualitative measures of stigma reduction.
2. Increase suicide prevention efforts in the community of farming and explore the relationship between change in self-stigma and perceived-stigma of suicide, suicide literacy, the nature of experience of suicide, age and health behaviour measures

## Methods

The Ripple Effect digital intervention [41] was developed to reflect defining characteristics of Australian rural farming communities as previously described by Brumby [42] and Kennedy [9]. The intervention was informed by adult learning models, empowering participants as peer agents of change, and providing opportunity to share insights, reflect, learn and apply new knowledge among people with shared interests, experiences and cultural context [43, 44]. Content was personalised and tailored to participants’ farming type (i.e. cattle, dairy, cropping), gender, and experience of suicide. This included personal stories through postcard messages, video stories, and education and personal goal setting. Content selection was informed by Corrigan’s social cognitive model of stigma, focusing on encouraging disclosure, building social connection, changing attitudes, improving awareness, and empowering participants through knowledge [45]. This approach has been successful in reducing self- and perceived- mental health stigma [27]. Given the paucity of evidence relative to suicide stigma reduction, parallels were drawn from this related body of research.

Recruitment to the intervention took a multifaceted approach and was shaped by the knowledge that people in Australia’s farming communities demonstrate a strong willingness to provide help to others, while avoid seeking help themselves [9]. Partners and stakeholders with links to the farming community were engaged to assist in sharing information about the Ripple Effect across their rural networks. This included industry newsletters, presentations at community, rural sporting club and farming industry gatherings and wide dissemination of information flyers through stakeholder networks. A Community Champions Network was developed to educate interested community members about the project—each member was provided a communications pack and regular project updates to assist them to share information about the intervention across their personal and professional networks. Social media platforms were set up on Facebook and Twitter and traditional media interest was harnessed to promote the project and support recruitment via regional and national television, radio, print and online media.

Given the nature of the study, attracting and retaining a true control group that would not seek information around suicide issues was seen as neither feasible nor ethical. Given this, it was decided not to include a control group. Despite the focus on a target male population (aged 30–64 years)—and the targeted recruitment campaign to support this--the broad nature of the impact of suicide in rural areas resulted in the decision to allow participation by all adults (male and female), thus avoiding the possibility of harm by exclusion. Restricting participation was viewed as unethical with potential to increase stigma and associated suicide risk.

The Ripple Effect digital intervention was divided into five discrete chapters and email reminders were sent at predetermined time points to encourage completion. Pre- and post-intervention assessment included:

- Suicide stigma: Self- and perceived-stigma were measured using an adapted form of the validated Stigma of Suicide Scale (SOSS) [46]. All participants were assessed for perceived-stigma. Participants who identified as having attempted suicide or experienced suicidal ideation were also assessed for self-stigma.
- Suicide literacy: Literacy was measured using the validated Literacy of Suicide Scale (LOSS) [47].

Qualitative data were collected at set progress points through the intervention. These included optional digital postcards inviting participants to provide personal insights, and opportunities to set personal goals relative to reducing stigma and improving mental wellbeing. Emailed invitations were sent to participants to return to the intervention and report on goal achievement.

All completing participants were provided opportunity (via an emailed link) to complete an online feedback survey comprising 16 qualitative and quantitative questions. Ethics approval was granted through Deakin University Human Research Ethics Committee (DUHREC) (2015–136).

A detailed description of the intervention can be viewed in the Ripple Effect Research Protocol [41].

### Data analysis

Quantitative data analysis was conducted using SPSS version 23 (IBM Corp., 2015). Analyses were undertaken on the full analysis set (FAS)—including all registered participants—applying the intention to treat principle (ITT). This was complemented with analyses of a per protocol set (PPS) that only included those completing the intervention and served as a sensitivity analysis. Missing values were accounted for using a mixed model analysis (residual maximum likelihood, REML) method. The null hypothesis—that there is no difference between assessment time periods—was conducted at the 5% significance level (α = 0.05).

Qualitative data was thematically analysed using the guidelines of Braun and Clarke [48]. Three members of the research team identified the themes. Data was then independently analysed by at least two researchers, with any discrepancies resolved through discussion within the research team.

Survey feedback data was collated and descriptively analysed.

## Results

**Fig. 1:**
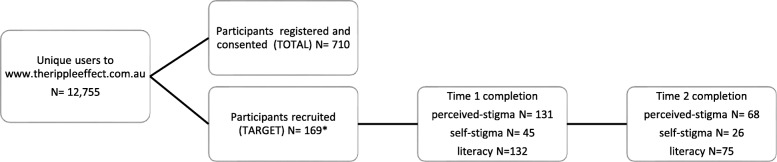
Intervention participation and target completion rates (*Target participants comprised those who had attempted to take their own life (*n* = 16); those who had thoughts about taking their own life (*n* = 37); those bereaved by suicide (*n* = 79); those who had cared for someone who attempted to take their own life (*n* = 6); and, those touched by suicide in some other way (*n* = 31))

During the research period (07/2016–01/2018), the Ripple Effect website had 12,755 unique visitors. Overall recruitment to the digital intervention exceeded the goal set in the Research Protocol (*n* = 473) [41]. Of the 710 participants who consented, 461 (64.9%) were female and 238 (33.5%) were male (five participants did not nominate a gender). The mean age of females was 41 years (standard deviation 14 years) and for males was 44 years (standard deviation 15 years). The largest category of response when asked what their farming enterprise was ‘Never farmed’ – 203 (44%) and 79 (33.2%) for females and males respectively. Of those who were farming, the most commonly represented industry.

was cattle farming (*n* = 56, 12.1%) and sheep farming (*n* = 35, 14.7%) for females and males respectively. The *n* = 169 used for the analyses were the target group which included males aged between 30 and 64 years of age. Figure 1 outlines participation and completion rates of the target group. Of those recruited from the target group, 131 completed the perceived-stigma assessment at Time 1 and 68 at Time 2. Fourty-five participants completed the self-stigma assessment at Time 1 and 26 at Time 2 (Note: Given the characteristics of self-stigma, opportunity to complete self-stigma assessment was only offered to those identifying as having attempted suicide (*n* = 16) or having previously had thoughts of attempting suicide (*n* = 37)). One hundred and thirty-two of the 169 recruited participants completed the suicide literacy assessment at Time 1 and 75 at Time 2.

**Fig. 2:**
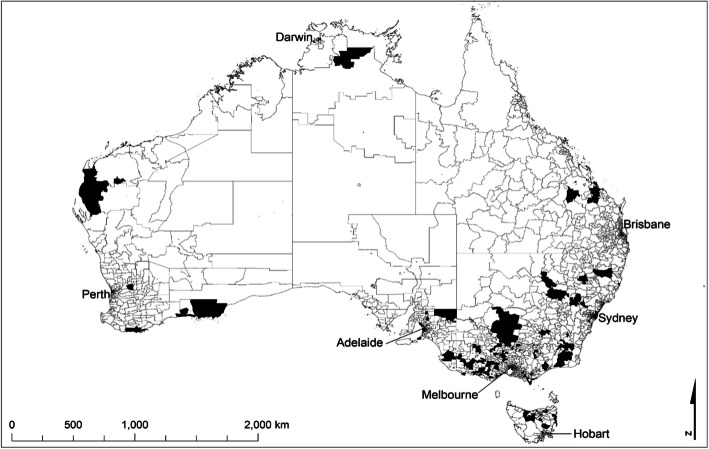
Target participant location by postcode (Note: (i) Named capital cities highlight the rural distribution of participants. Remote Australia has vast areas represented by a small number of postcodes, therefore not necessarily representing high participant numbers; (ii) The authors, using publically available data [49], created the map using ArcGIS® ArcMap™ software [50]. All of Australia’s postcodes are presented with the project’s reach indicated by highlighted areas)

**Fig. 3:**
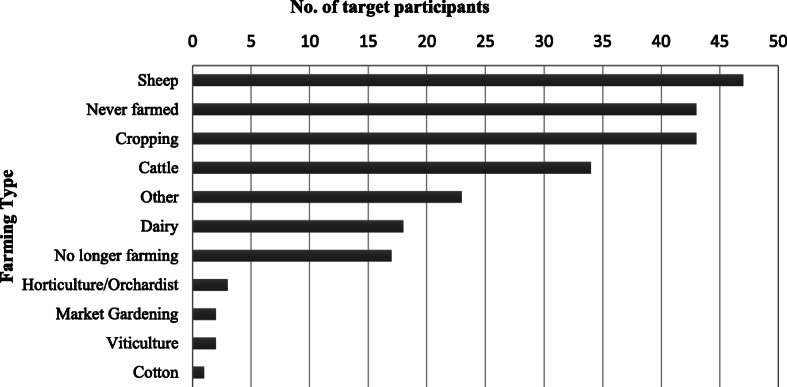
Farming type of target participants (*n* = 169) (Note: Total number of farming type is greater than the number of participants as participants were frequently involved in more than one type of farming industry)

The intervention was successful in reaching members of the target group from across Australia’s rural communities—with negligible participation in capital cities—representing diversity of both geographic location (see Fig. 2) and farming industry (see Fig. 3). There were *n* = 166 target participants with valid postcodes, with representatives from *n* = 129 postcodes across Australia. The majority of postcode areas (*n* = 108) were represented by at least one participant (*n* = 108, 84%), with a maximum of five participants from any one postcode area (*n* = 2).

### Change in stigma as identified via validated (adapted) stigma scale

For both self-stigma and perceived-stigma adaptions of the Stigma of Suicide Scale (SOSS), there was no significant change from Time 1 to Time 2 on the stigma subscale on either the FAS or the PPS (see Table 1). Similarly, there was no significant change for the isolation/depression subscale. On the glorification/normalisation subscale there was a significant increase from Time 1 to Time 2 for perceived-stigma only, suggesting that participants increased their belief that *other* people would be likely to glorify/normalise suicide, whilst not increasing their own belief in the glorification/normalisation of suicide.

**Table 1:** Predicted means (standard errors) of SOSS (self and others) for target group participants in the Ripple Effect

FAS^a^	Time 1 (T1)	Time 2 (T2)	Difference (T2-T1)	*P*-value
*Stigma of Suicide Scale: perceived-stigma subscale*				
Stigma	23.07 (0.54)	23.34 (0.71)	0.27	0.713
Isolation-Depression	14.54 (0.27)	14.13 (0.34)	− 0.41	0.222
Glorification-Normalisation	7.89 (0.22)	8.24 (0.25)	−0.35	0.038*
PPS^b^				
Stigma	22.46 (0.76)	23.10 (0.76)	0.64	0.411
Isolation-Depression	14.19 (0.40)	13.99 (0.40)	−0.21	0.562
Glorification-Normalisation	7.69 (0.32)	8.03 (0.32)	−0.34	0.045*
*Stigma of Suicide Scale: self-stigma subscale*				
Stigma	22.38 (1.05)	21.32 (1.19)	−1.06	0.242
Isolation-Depression	15.09 (0.70)	13.74 (0.85)	−1.34	0.101
Glorification-Normalisation	8.07 (0.50)	8.94 (0.58)	0.88	0.078
PPS^b^				
Stigma	20.72 (1.56)	20.23 (1.57)	−0.49	0.612
Isolation-Depression	14.96 (1.00)	13.38 (1.02)	−1.58	0.094
Glorification-Normalisation	7.48 (0.71)	8.48 (0.71)	1.00	0.070

*p < 0.05

^a^Full Analysis Set

^b^Per Protocol Set

### Changes in stigma as identified via behavioural indicators

#### Digital postcard messaging

At four time points during the intervention, participants were invited to complete a digital postcard and share their insights about their experience of suicide, their experience of talking about suicide, their personal strategies in relation to their experience of suicide, and how their thoughts of suicide had been affected by participating in the intervention. Target participants submitted a total of 78 digital postcards. The majority of postcards conveyed positive examples of behaviours geared towards stigma reduction or encouraging stigma-reducing behaviours in others.

Ten postcard messages conveyed participants’ belief that their feelings about suicide had not changed as a result of participating in the Ripple Effect. While three of these messages offered no further explanation, the remainder included some form of clarification. Some of these messages went on to indicate a confirmation of non-stigmatising knowledge, like one suicide-bereaved participant who recognised his response to loss was not abnormal: “[My thoughts about suicide] probably haven’t changed, just provided clarity that my thoughts are normal” (Male, 57 years). Another of these participants acknowledged his continuing (but unchanged) belief in the value of support: “[My thoughts about suicide have] not really [changed]. If not for the love from my wife ... cats and dog I would have stepped off planet ages ago” (Male, 47 years). Several of these went on to include a positive message—often including indicators of behaviour directed to reduce stigma:

I don’t think my thoughts have changed. I continue to recognise suicide is a much deeper mental health challenge than most people realise. It’s important that more people take steps to understand that no matter who you might be in society you can be vulnerable but likewise, you can help others. (Male, 58 years).

Even when not recognising any personal change, participants recognised the value of the intervention in encouraging conversations about suicide: “This hasn’t changed my thoughts about suicide, but it is good to see this project encouraging conversations” (Male, 60 years).

Several participants’ postcards showed behaviours suggestive of stigma reduction as a direct result of their participation in the intervention. When prompted to write a postcard in response to the question ‘Have your thoughts about suicide changed as a result of participating in the Ripple Effect?’ participants spoke about a renewed focus on self-care: “The Ripple Effect has helped move me into a new phase of my on-going maintenance of my mental health” (Male, 34 years).

Other participants described a reduction in self-stigma and a greater willingness to seek support: “I no longer believe it is embarrassing- and it is that past thought that stopped me reaching out [ …] I am not pretending, I am suffering. There is nothing embarrassing about that. In fact, it is brave to reach out” (Male, 62 years).

The majority of postcards contained messages about the personal suicide experiences of participants, irrespective of prompt questions. Four subthemes were identified relative to personal experience including ‘growth’, ‘new realisations’, ‘hope’ and ‘encouragement’. Within these themes were indications of attitudinal and behaviour change indicative of reduced stigma associated with mental health and suicide.

##### Growth

Numerous postcards shared messages indicating stigma reduction as a component of post-traumatic growth that occurred following an experience of suicide (Tedeschi & Calhoun, 2007). These participants often expressed previous feelings of isolation and judgement (by self and others) that had changed over time into a desire to support others and recognise the value of self-care. As one participant explained:

My thoughts have changed. At the time I was sad, no one but me knew what I had been through in my life, some didn’t want to listen it was unbelievable to them. Others couldn’t listen they didn’t have the time, most could have helped but choose not to as they didn’t know how. Now I am stronger having lived through the hardest times of anyone’s life, I see people struggling and know exactly how to raise issues and talk to others through my life experiences. I don’t care so much about what people think and focus on my inner peace and development. (Male, 56 years).

##### New realisations

While post-traumatic growth was often inferred as occurring over time, participants also highlighted particular moments or events when a turning point had been reached. These instances highlighted the reduction of stigma. One example highlighted the relief experienced from seeking support, and the ongoing benefit of communicating emotions:

The first time I went and saw a counsellor, it felt like the dam wall broke. Everything I had bottled up inside of me just flooded out and I walked out of there 20 k lighter than when I walked in. It also opened the gates for further communication about my feelings, which I had previously not done much. (Male, 61 years).

Another example highlighted the turning point resulting from a friend’s suicide death, and the shift from harmful coping strategies to a more positive focus:

Dealing with my friend’s suicide was a huge prompt/challenge for me to better manage my own mental health and wellbeing. I have progressed from escaping through drugs to using yoga and meditation now to realise more health and happiness and meaning in life than I ever thought possible. (Male, 36 years).

##### Hope

Not all participants had transitioned from awareness and attitudinal change to behaviour change. This example highlights the aspirational, yet not fully realised, stigma reducing behaviours of one participant: “I just need to manage myself better when I have these thoughts. Concentrate on exercise and social interaction and understand that I will come out the other side from this difficult time” (Male, 64 years).

##### Encouragement

A resounding message throughout the postcards was participants’ wish to pass on the learnings from their own experience to ease the burden of others to follow. Several messages contained emotional encouragement:

At the time I attempted to take my own life, my world had practically fallen apart around me […] I look back at those days now and I am so very thankful that none of my attempts were successful […] I’ve got a great support network around me and that takes the edge off things. The biggest piece of advice I can give is just don’t give up. It does get better. It’s not easy, but it does happen. (Male, 33 years).

Other messages also provided practical advice:

Keep talking to those you trust, seek medical help and see a counsellor. Don’t stop talking about how you feel and your emotions, don’t be afraid to cry and let it out! Look after yourself with regards to diet, exercise, and keep those close to you who mean the most such as your kids. (Male, 31 years).

The majority of postcard messages shared included acknowledgement of pain and loss juxtaposed with messages of encouragement, personal growth, hope and accompanying indications of stigma reducing behaviours.

#### Personal goal setting

**Fig. 4:**
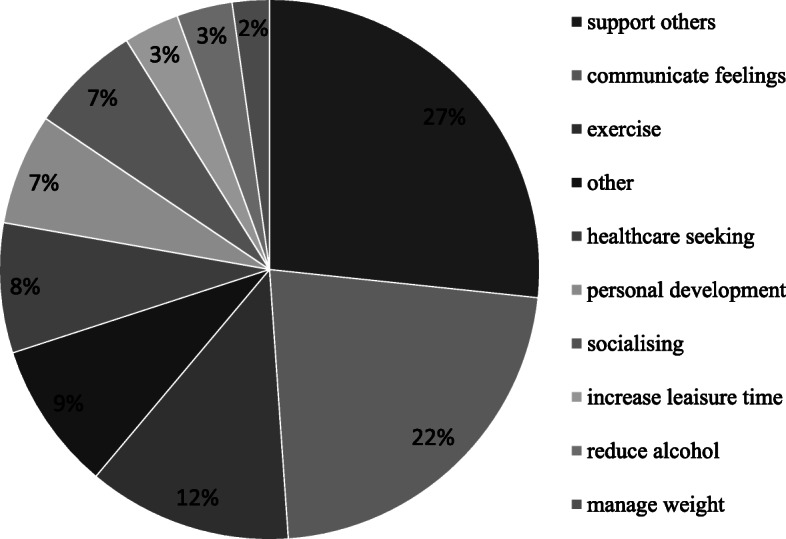
Personal goal setting (*n* = 90)

Although personal goal setting was optional, 70 participants set a total of 90 goals. Thematic analysis of the goals identified behavioural indicators of stigma reduction across a number of areas (see Fig. 4) including supporting others (*n* = 24), communicating feelings (*n* = 20), seeking healthcare support (*n* = 7) and social connection (*n* = 6). Participants also made the connection between their mental wellbeing and physical health and set goals around physical activity (*n* = 11), and weight management (*n* = 2). Encouragingly, a small number of participants also committed to reducing alcohol use (*n* = 3)—a known risk factor for suicide. Despite email reminders, response rates about goal achievement were very poor with only two participants reporting back.

### Changes in suicide literacy

As outlined in Table 2, participants’ baseline suicide literacy was high when compared with previous community samples [47]. Some improvement over time was identified, in both the ITT and PPS. The PPS had a greater degree of improvement in suicide literacy over time—suggesting that participants finishing the intervention had a greater improvement in suicide literacy than those who did not complete—although this did not reach statistical significance.

**Table 2:** Change over time for TARGET group in mean total Literacy of Suicide Scale (LOSS) score^a^

FAS^b^	T1	T2	Difference (T2-T1)	*P*-value
LOSS	9.68 (0.16)	9.80 (0.21)	0.12	0.571
PPS^c^	T1	T2	Difference (T2-T1)	*P*-value
LOSS	9.82 (0.22)	10.07 (0.22)	0.25	0.254

^a^Mean number of items correct out of 12 items in total. Based upon those who completed the intervention and responded to the LOSS at T1 and T2 (*n* = 67)

^b^Full Analysis Set

^c^Per Protocol Set

The two lowest scoring items on the LOSS [47] at T1 for the target group related to knowledge linking suicide risk and alcohol use (*There is a strong relationship between alcoholism and suicide)* and the potential to change one’s mind about suicide (*People who want to attempt suicide can change their mind quickly*). These two items were the only areas of suicide literacy with either significant improvement (*p* < 0.05 for alcoholism and suicide) or improvement approaching significance (*p* = 0.053 for changing mind).

### Participant feedback following completion of the intervention

Twenty completing participants completed an online feedback survey (see Table 3). Respondents represented the range of suicide experience (attempted suicide, thoughts of suicide, bereaved by suicide, carer of someone who attempted suicide, touched by suicide in another way). Given the survey was anonymous and not linked to intervention data, it was not possible to distinguish target participants.

**Table 3:** Responses to post-completion feedback survey (*n* = 20)

*Survey item*	*Percentage of feedback survey respondents*
First time I have shared my experience publically	60
Importance of anonymous contribution	85
Participation elements	
Set one or more personal goals	85
Completed one or more digital postcards	80
Improved understanding	
Suicide stigma and how this may be overcome	67
Risk and protective factors and tipping points for suicide	63
Complexity of contributing factors	74
Benefits of safe conversations about suicide	74
Increased skills	
How to support own and others’ wellbeing	80
More likely to have a conversation about experience	65
More likely to engage with formal services	74
More likely to engage with informal services	68
Helpful elements of intervention	
Digital stories	95
Written information	95
Digital postcards	84
Geographically personalised list of resources	74
Navigational features of intervention	89
Valued peer-based design of intervention	
Importance of shared understanding of farming life/work	90
Importance of shared understanding of suicide experience	95

## Discussion

### Suicide stigma

When measured by the Stigma of Suicide Scale, the intervention demonstrated no significant reduction in stigma over time. However, there were notable behavioural indicators of stigma reduction. This was demonstrated by participants through goal setting and shared postcard messaging. Encouraging indicators highlighting participants’ readiness to support others, initiate challenging conversations, increase social connection, and seek support suggest that stigma has, in fact, reduced [51]. The high rate of personal goal setting reflects the practical, goal-directed focus identified within farming community members in previous research [9, 52]. A strong focus on supporting others is not surprising given evidence highlighting people from rural farming communities’ readiness to offer support to others [9]. Traditionally, this has been coupled with a reticence from farming community members—both males and females—to seek support themselves [9]. Encouragingly, behavioural indicators identified by patterns of goal setting and through digital postcards do not necessarily support a reticence for seeking support per se, despite goals associated with offering support outweighing goals for seeking professional healthcare support. Goals reflecting engagement in peer-based support were reflected in ‘communicating feelings’ and ‘social connection’. This is encouraging given previous research indicating stigma as a previously identified barrier to even discussing mental health concerns with others [53]. It could be that the shared understanding demonstrated by peers may encourage support seeking—supporting previous research suggesting farmers are interested in looking after their health and wellbeing, if the context of engagement reflects an understanding of farming life and work [42]. It could also be that factors other than stigma—including poor knowledge of the mental health system, confusion about available services, cost concerns and lack of care coordination—minimised participants’ goal-setting and postcard messages about seeking professional mental health support [54]. This reinforces the importance of professional support services to be physically accessible, as well as demonstrating an understanding of farming life and work, and delivering services that are relevant within rural contexts.

Given the small number of participants who provided feedback on achieving goals set, it is difficult to determine whether intentions were translated into action. Further work is required to determine whether indications of stigma reduction in personal goals and postcard messages translate into behaviour change, although previous research with farmers has shown personal goals around health and wellbeing are translated into action [52]. More direct and sustained follow-up with participants to evaluate goal setting, goal achievement, and goal outcomes would be valuable. Reminders sent via Short Message Service (SMS or text message)—rather than email—may be a more direct and engaging way of eliciting responses. Health interventions have identified positive response rates when using text message reminders, particularly when these reminders are tailored to the participant and require the participant to respond [55]—both of which are possible within the current intervention platform. Social media platforms, such as WhatsApp and Messenger, should also be considered as additional methods of engagement.

The increase in the perceived-stigma glorification/normalisation subscale over time can be interpreted in multiple ways. Repeated exposure of participants to personal stories from people with an experience of suicide may have facilitated identification/empathy with other farming community members with similar experiences. What could be considered as ‘normalisation’ in this case, is an expected—and encouraged—product of de-stigmatisation via interpersonal contact [56]. In this situation, increased normalisation would be interpreted as a positive outcome. Given that the video stories frequently highlighted personal strength and post-traumatic growth developed by people with lived experience of suicide, it may also be that participants’ increased levels of glorification were reflecting a belief that those with a lived experience of suicide would be perceived by others as ‘strong’, ‘noble’, ‘brave’ and ‘dedicated’ (the statements by which the glorification/normalisation subscale were assessed), rather than glorifying the act of suicide itself (what the original SOSS measures). While glorification of suicide is to be eschewed, glorification of people who have the strength to overcome suicide ideation, seek support or use their experience to help others should be considered a positive, de-stigmatising outcome. Perceptions of normalisation and glorification—in the context of lived experience, as opposed to the act of suicide—requires further research.

### Suicide literacy

Participants demonstrated high baseline suicide literacy when compared with previous community samples [47]. This is not surprising as the intervention actively sought out target males with experience of suicide from rural areas (where there is also a higher exposure to suicide)—resulting in participants with first-hand knowledge and increased awareness of the context in which suicide occurs. Consequently, increasing literacy as a pathway to reducing stigma was challenging as the baseline levels for most literacy items were already very high—creating a possible ceiling effect for this sample. While not statistically significant, greater improvements for the Per Protocol Set (when compared with Intention To Treat) suggests that participants completing the intervention had a greater improvement in suicide literacy levels than those who did not complete. Further improving suicide literacy in such an informed community—with a view to reducing stigma and preventing suicide—is likely to require targeted, context-specific information not related to increasing literacy per se or as currently measured by the LOSS. This may include information about the link between suicide and alcohol misuse—an area of knowledge identified as deficient in the target group and previously identified as a concern within farming communities [57]. This may also require new ways of assessing suicide literacy. The link between improving literacy, reducing stigma and preventing suicide must also be examined in greater detail, given that the target male group demonstrated very high literacy, yet represent the population with the greatest proportion of suicides in rural farming areas [5].

### Recommendations for improving understanding of suicide stigma and literacy within rural Australia

Participants demonstrated differences in levels of stigma and suicide literacy when compared to previous general community samples [58]. Therefore, more detailed qualitative research is required to appreciate (i) how suicide is understood, and (ii) how stigma is communicated, experienced and maintained in Australian rural and remote and farming communities. This exploration should include the target group (males aged 30–64 years) as well as males outside the target group and females.

Outcomes of participation in the intervention do not adequately capture the level of community engagement and likely stigma reduction associated with this across rural Australia. The Ripple Effect intervention had engagement with over 450 stakeholders, supported over 60 ‘community champions’ who voluntarily spread the message of stigma reduction across Australia, and generated a social media reach of over 400,000 people [59]. This raises a number of challenges including (i) how to more effectively drive social media traffic to participate in the digital intervention, and (ii) how to meaningfully describe and evaluate stigma change outside of—but linked with—the intervention.

### Recommendations for improving suicide prevention in Australia’s rural farming population

This research has demonstrated high levels of suicide literacy and high levels of community will to improve rural mental health and prevent farmer suicide. However, farmer suicide continues to be a concern. Previous research suggests that farmers are likely to have a rapid suicide trajectory, given their access to lethal means [6] and their acclimatisation to risk taking behavior [60]. The authors propose the development of innovative methods to prevent farmer suicide, focused on developing interventions occurring rapidly during a moment of crisis. This could build on the concepts underpinning suicide safety planning [61] in such a way that reflects the context and specific suicide risks farmers face as well as the protective factors that are available to them.

### Strengths and limitations

This intervention had a number of strengths. Firstly, collaborating with a range of stakeholders (including farmers and industry bodies) provided valuable opportunities for engagement and recruitment of participants from the farming community across Australia. Secondly, the development of a sophisticated web-based platform meant that content and engagement were able to be tailored to each participant’s context including the imagery, digital stories and framing of the information presented to each participant. In combination, these two strengths resulted in the intervention being able to engage with a population that is particularly vulnerable to stigma and suicide risk [4–7, 9] and a population that are considered different to the general community samples on which stigma research has traditionally focused.

This intervention also had several limitations. Given the nature of a digital intervention, participation was limited to those who had access to online communications—a concern that is decreasing, yet still present across some areas of rural Australia.

This is the first intervention to use an adapted form of the SOSS [46] and to use the SOSS pre- and post-intervention. This limits comparability with previous samples and the resulting interpretation of findings.

Given the recruitment method employed in this study (invited participation by those who self-identified as having been affected by suicide) we are unable to definitively claim the sample is representative of our target group. Recent international evidence suggests that at least 135 people are affected by every suicide death [62]. In rural farming communities, anonymity is low and tight-knit social connections prevail. Therefore, it is reasonable to assume that most people in our target group would have been affected by suicide in some way, and this inclusion criterion wouldn’t necessarily lead to an unrepresentative sample.

While behavioural indicators of stigma reduction were indicated by personal goal-setting tasks, the feedback from participants regarding goal attainment was minimal. Improved and innovative digital strategies for maintaining engagement and encouraging feedback are required to establish the translation of intent to action.

## Conclusions

Improving communication about rural suicide helps individuals with lived experience of suicide, and assists researchers, health practitioners, and policy makers develop appropriate and more effective evidence-based responses. Encouraging open and safe communication will also counter the thought and behaviour patterns that maintain personal and structural stigma in rural communities. The previously identified evidence of association between increasing mental health literacy and decreasing mental health stigma may not apply as aptly to suicide literacy and suicide stigma–as found in this population. Therefore, future research must ensure that the link between improving literacy, reducing stigma and preventing suicide is examined in both greater and closer detail—as demonstrated in this highly literate group. This is a vital consideration for any future suicide prevention work.

## Data Availability

The datasets used and/or analysed during the current study are available from the corresponding author on reasonable request.

## Abbreviations

SOSS: Stigma of Suicide Scale
LOSS: Literacy of Suicide Scale
PPS: Per-Protocol Set
FFS: Full Analysis Set
T1: Time 1
T2: Time 2
